# Automated processing of thermal imaging to detect COVID-19

**DOI:** 10.1038/s41598-021-96900-9

**Published:** 2021-09-01

**Authors:** Rafael Y. Brzezinski, Neta Rabin, Nir Lewis, Racheli Peled, Ariel Kerpel, Avishai M. Tsur, Omer Gendelman, Nili Naftali-Shani, Irina Gringauz, Howard Amital, Avshalom Leibowitz, Haim Mayan, Ilan Ben-Zvi, Eyal Heller, Liran Shechtman, Ori Rogowski, Shani Shenhar-Tsarfaty, Eli Konen, Edith M. Marom, Avinoah Ironi, Galia Rahav, Yair Zimmer, Ehud Grossman, Zehava Ovadia-Blechman, Jonathan Leor, Oshrit Hoffer

**Affiliations:** 1 Neufeld Cardiac Research Institute, Sackler Faculty of Medicine, Tel Aviv University, Tel Aviv, Israel; 2 Tamman Cardiovascular Research Institute, Leviev Heart Center, Sheba Medical Center, 52621 Tel Hashomer, Israel; 3 Faculty of Engineering, Tel-Aviv University, Tel Aviv, Israel; 4 Department of Diagnostic Imaging, Sheba Medical Center, Tel Hashomer, Israel; 5 Sackler Faculty of Medicine, Tel Aviv University, Tel Aviv, Israel; 6 Internal Medicine B, D, E, and F, Sheba Medical Center, Tel Hashomer, Israel; 7 Israel Defense Forces, Medical Corps, Ramat Gan, Israel; 8 Geriatrics Division, Sheba Medical Center, Tel Hashomer, Israel; 9 Internal Medicine C, D, and E, Tel Aviv Sourasky Medical Center, Tel Aviv, Israel; 10 Department of Emergency Medicine, Sheba Medical Center, Tel Hashomer, Israel; 11 Infectious Disease Unit, Sheba Medical Center, Tel Hashomer, Israel; 12 School of Medical Engineering, Afeka Tel Aviv Academic College of Engineering, Tel Aviv, Israel; 13 Internal Medicine Wing and Hypertension Unit, Sheba Medical Center, Tel Hashomer, Israel; 14 School of Electrical Engineering, Afeka Tel Aviv Academic College of Engineering, Tel Aviv, Israel

**Keywords:** Infectious diseases, Medical imaging

## Abstract

Rapid and sensitive screening tools for SARS-CoV-2 infection are essential to limit the spread of COVID-19 and to properly allocate national resources. Here, we developed a new point-of-care, non-contact thermal imaging tool to detect COVID-19, based on advanced image processing algorithms. We captured thermal images of the backs of individuals with and without COVID-19 using a portable thermal camera that connects directly to smartphones. Our novel image processing algorithms automatically extracted multiple texture and shape features of the thermal images and achieved an area under the curve (AUC) of 0.85 in COVID-19 detection with up to 92% sensitivity. Thermal imaging scores were inversely correlated with clinical variables associated with COVID-19 disease progression. In summary, we show, for the first time, that a hand-held thermal imaging device can be used to detect COVID-19. Non-invasive thermal imaging could be used to screen for COVID-19 in out-of-hospital settings, especially in low-income regions with limited imaging resources.

## Introduction

The coronavirus disease 2019 (COVID-19) pandemic has imposed an immense burden on out-of-hospital health care services and community-based testing sites worldwide^1,2^. Immediate and sensitive screening tools for severe acute respiratory syndrome coronavirus 2 (SARS-CoV-2) infection are essential in order to limit the spread of the disease and to properly allocate national resources. The common and standard diagnosis of SARS-CoV-2 infection is based on a virus-specific quantitative real-time PCR (qRT-PCR). The fastest test, however, can take up to several hours to complete, depending on available resources and disease burden in the region^3^. Furthermore, a significant amount of skilled labor is required at the different stages of qRT-PCR testing (sampling, preparation, and analysis)^3^.

Infrared thermography scanners have been widely used as a screening tool during past infectious disease epidemics, including COVID-19^4,5^. This technique is based on the assessment of absolute body temperature in order to screen individuals with fever, and lacks specificity for COVID-19^5^.

Non-invasive thermal imaging of organ-specific diseases has been presented as a new tool to detect inflammation and vascular dysfunction^6–8^. The use of thermal imaging has been demonstrated in the monitoring of various diseases such as breast cancer, diabetic neuropathy, kidney transplant defects, dermatological disorders, chronic wound treatment and more^9–11^. We propose that advanced processing of thermal images, combined with machine learning analysis, could serve as an innovative diagnostic tool for detection of COVID-19 and its associated microvascular injury.

Here, we developed automated image processing algorithms for analysis of thermal images of the back, captured by a portable thermal camera that connects directly to smartphones. We aimed to determine the ability of this novel technique to detect COVID-19-associated lung injury. Our automated thermal imaging tool is relatively cheap, easy to use, and delivers immediate test results. It is therefore especially applicable for out-of-hospital screening by first responders (paramedics, nurses, medical technicians, etc.), home care facilities, military bases, and regions with limited diagnostic resources.

## Results

### Study cohort

The final study cohort included 101 individuals who were prospectively enrolled from two medical centers in Israel (Table 1). The mean age was 56 ± 17 years, and 80 participants (79%) were men. A total of 62 (61%) individuals were COVID-19 positive, of whom 40 (85%) had some form of lung injury, defined as any clinical or radiological evidence of pneumonia or acute respiratory distress, including hypoxemia, dyspnea, respiratory rate > 20, shortness of breath, severe cough, and/or the presence of ground-glass appearance, consolidation, or linear opacities on chest X-ray (CXR)^12^.

**Table 1:** Patient characteristics.

Characteristic	COVID-19 negative	COVID-19 positive	p
	n = 39	n = 62	
Age, years	48.8 ± 20	60.6 ± 13	0.01
Sex (male), n (%)	31 (80%)	49 (79%)	0.9
Lung injury, n (%)	7 (18%)	40 (65%)	0.01
Body temperature, °C	37.1 ± 0.5	36.9 ± 0.6	0.3
SPO_2_ room air, %	95.7 ± 2.9	94.4 ± 4.2	0.3
Hypertension, n (%)	10 (26%)	24 (39%)	0.2
Dyslipidemia, n (%)	10 (26%)	28 (45%)	0.05
Diabetes, n (%)	6 (15%)	15 (24%)	0.3
CRP, mg/L	14.1 [2.0, 96.5]	47.9 [11.8, 120.4]	0.5
D-dimer, mg/mL	401 [187, 792]	503 [174.1, 745]	0.4
WBC, 10^9^/L	8.7 [7.1, 10.9]	6.3 [5, 7.9]	0.06
Cardiac troponin-I, ng/L	3.3 [2.7, 13.3]	6.2 [4, 12.1]	0.7
Hemoglobin, g/dL	12.2 ± 2.5	13.6 ± 2.3	0.03

Values are presented as mean ± SD, or median [interquartile range] for irregularly distributed parameters. Blood test results were taken from the day of imaging (n = 78).

* CRP* C-reactive protein,* WBC* white blood count,* SPO*_2_ saturation of peripheral oxygen.

P values by two-tailed unpaired t-test, non-parametric Mann-Whitney test, and Chi-square test.

Patients with COVID-19 were older and had a higher prevalence of lung injury than patients without COVID-19 (Table 1, 65% vs. 18%, p < 0.01). Absolute body temperature, as well as the prevalence of hypertension, dyslipidemia, and diabetes, were not different between the groups (Table 1). Overall, the majority of COVID-19 patients included in our study were ultimately discharged with a favorable prognosis. Only three patients required mechanical ventilation during follow-up; two eventually died (one with and one without COVID-19).

### Thermal image processing detected COVID-19 and lung injury status

**Figure 1:**
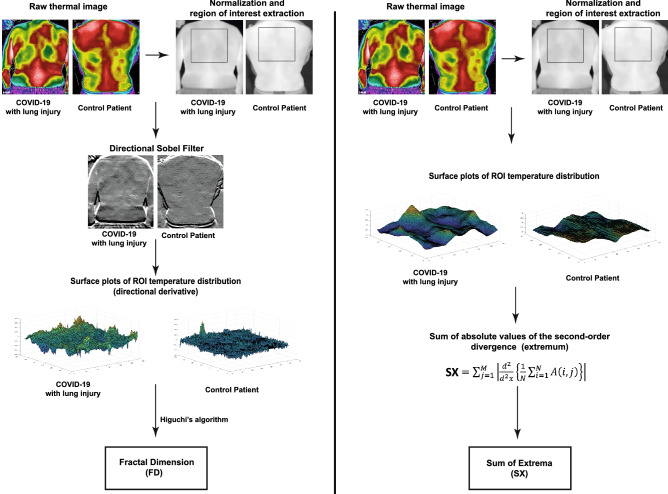
Thermal image processing. The raw thermal images are displayed in HC-Rainbow scale. Displayed are representative images of the different stages of our image processing algorithms for the fractal dimension (FD) of the gradient (left) and the “sum of extrema” (SX) in the image (right). For this display the gradient range [-8, 8] was normalized to [0, 255]. All gradient values outside of this range were shifted to its edges before normalization. Full details on image processing algorithms are provided in the Methods section. *ROI *region of interest.

**Figure 2:**
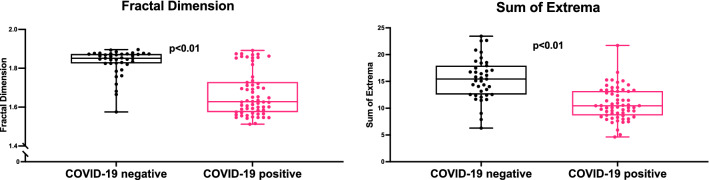
Thermal imaging scores according to COVID-19 disease status. Box plots with individual values of fractal dimension (FD) (left side) and sum of extrema (SX) (right side) scores. Patients who were COVID-19 positive had lower thermal scores compared to COVID-19 negative individuals. P value by unpaired t-test.

We captured thermal images of the participants’ exposed upper backs (over the lungs) using a portable thermal camera that connects directly to smartphones. All images of the final study cohort (n = 101) were analyzed by investigators who were blinded to the patients' clinical data. Our novel thermal image processing algorithms extracted advanced texture and shape features of temperature distribution across the skin. Two key parameters were chosen for downstream analysis: the fractal dimension of the gradient (termed FD) and a new parameter we called the “sum of extrema” in the image (termed SX; see further details in the Methods section) (Fig. 1). Both FD and SX scores were significantly lower in COVID-19 positive individuals compared to the rest of the cohort (Fig. 2).

**Figure 3:**
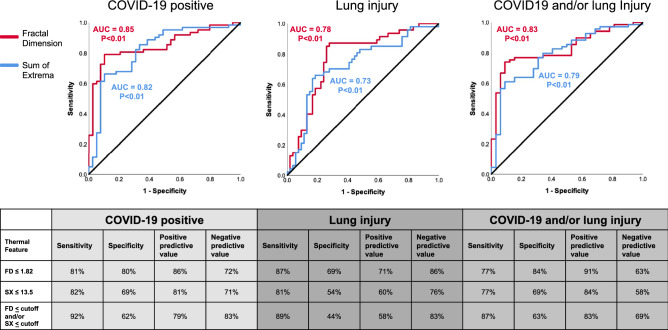
The diagnostic yields of thermal imaging scores in detection of COVID-19 and lung injury. Receiver operating characteristic (ROC) curves are shown (top panel) for the three different clinical classifications. Sensitivity and specificity values were calculated for select cutoffs (bottom panel).

Next, we evaluated the ability of our thermal scores to detect COVID-19 disease status and/or the presence of lung injury. Sensitivity, specificity, and area under the curve (AUC) were calculated for both FD and SX scores. The receiver operating characteristic (ROC) curves (Fig. 3) indicated that both FD and SX scores were significantly associated with the diagnosis of COVID-19, with an AUC of 0.85 (95% CI: 0.78, 0.93; p < 0.01) and 0.82 (95% CI: 0.73, 0.91; p < 0.01) respectively. A combined cutoff of a low FD score (≤ 1.82) and/or low SX score (≤ 13.5) demonstrated 92% sensitivity and 62% specificity in detection of COVID-19 status (regardless of lung injury) (Fig. 3). Thus, the two thermal scores FD and SX were able to identify COVID-19 in suspected individuals.

FD and SX scores were also significantly associated with lung injury, with an AUC of 0.78 (95% CI: 0.69, 0.88; p < 0.01) and 0.73 (95% CI: 0.63, 0.83; p < 0.01) (Fig. 3).

The combined cutoff of a low FD score and/or low SX score demonstrated a high sensitivity of 89% but a specificity of only 44% in detection of lung injury (regardless of COVID-19 status) (Fig. 3). FD outperformed SX in detection of the composite diagnosis of COVID-19 and/or lung injury, with an AUC of 0.83 (95% CI: 0.75, 0.91; p < 0.01) vs. 0.79 (95% CI: 0.70, 0.88; p < 0.01).

To determine the effect of the anatomical location of the selected region of interest (ROI) on diagnostic yields, we ran our algorithms on a different ROI: the lower back region, below the lungs (Supp Fig. 1). Both FD and SX scores demonstrated similar diagnostic yields for detection of COVID-19 status compared with the upper back ROI selection model: AUC = 0.85 (95% CI: 0.76, 0.93; p < 0.01) for FD and 0.79 (95% CI: 0.7, 0.88; p < 0.01) for SX. Thus, it seems that the differences in temperature distribution associated with COVID-19 are not restricted to the skin covering the lungs. We speculate that these changes may reflect a systemic pattern present across the entire torso.

### The correlation between thermal imaging scores and clinical variables

**Figure 4:**
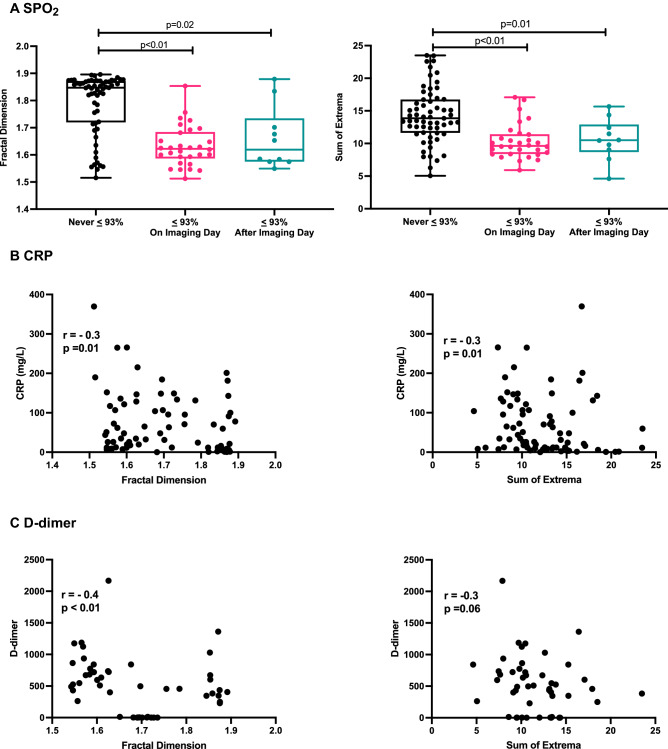
Thermal imaging scores and clinical measurements. (**A**) Box plots with individual measurements of the fractal dimension (FD) (left) and sum of extrema (SX) (right). P value by one-way ANOVA with Tukey’s test for multiple comparisons. (**B**), (**C**) The correlation between the thermal FD and SX scores and C-reactive protein (CRP) (**B**) and D-Dimer (**C**) measured on the day of imaging. P value by Spearman’s correlation test.

Next, we evaluated the correlation between our thermal scores and selected clinical variables that were shown to predict poor prognosis in COVID-19 patients^13,14^. Patients with saturation of peripheral oxygen (SPO_2_) ≤ 93% on the day of imaging had lower FD and SX scores than patients with normal SPO_2_: mean FD = 1.63 ± 0.07 vs. 1.79 ± 0.11, p < 0.01; mean SX = 10.29 ± 2.68 vs. 14.28 ± 4.22, p < 0.01 (Fig. 4A). Moreover, even patients who had normal SPO_2_ values on the day of imaging but developed hypoxemia (≤ 93%) during follow-up had lower FD and SX scores: mean FD = 1.66 ± 0.11 vs. 1.79 ± 0.11, p = 0.02; mean SX = 10.6 ± 3.19 vs. 14.28 ± 4.22, p = 0.01 (Fig. 4A). The latter finding suggests that thermal imaging may provide an early indication for clinical deterioration. In line with these observations, patients who required non-invasive oxygen support such as nasal cannula or face masks had lower FD scores than patients who did not need oxygen therapy: 1.67 ± 0.07 vs. 1.74 ± 0.13, p < 0.01. Overall, low blood oxygen saturation was associated with low FD and SX thermal scores.

C-reactive protein (CRP) and D-dimer levels are associated with COVID-19 severity and prognosis^13,14^. Significantly, the thermal FD and SX scores were inversely correlated with CRP and D-dimer levels measured on the day of imaging (Fig. 4B,C). We did not find a correlation between FD and SX with cardiac troponin-I concentrations: r = 0.04, p = 0.78, and r = -0.22, p = 0.11. Taken together, the thermal FD and SX scores were inversely correlated with clinical variables that reflect COVID-19 disease progression.

### Thermal imaging scores did not correlate with the findings on chest X-ray

Finally, to compare our thermal imaging technique to conventional CXR, we used the radiographic assessment of lung edema (RALE) score^15^. We compared thermal imaging scores with RALE scores for all patients who had a CXR within 6 h of thermal imaging (n = 30). The CXRs were manually reviewed by a certified radiologist who was blinded to the patients' clinical statuses and thermal imaging scores.

**Figure 5:**
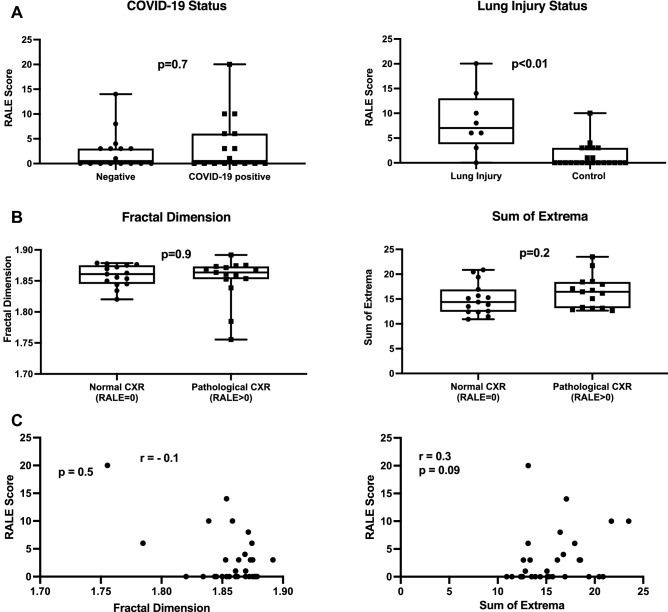
The correlation between thermal imaging and chest X-ray. (**A**) Box plots of RALE score according to COVID-19 and lung injury status. P value by Mann–Whitney test. (**B**) Box plots of the fractal dimension (FD) (left) and sum of extrema (SX) (right) thermal scores according to categorized RALE score. P value by Mann–Whitney test. (**C**) RALE scores were not correlated with thermal imaging scores. P value by Spearman’s correlation test. *CXR* chest X-ray, *RALE* radiographic assessment of lung edema.

First, in line with a previous report^15^, RALE scores were similar in patients with and without COVID-19, and did not provide diagnostic yields (Fig. 5A). As expected, patients with lung injury had higher RALE scores than control patients (Fig. 5A). However, patients with a pathological CXR (RALE score > 0) and patients with a normal CXR (RALE score = 0) had similar FD and SX scores (Fig. 5B). Both thermal FD and SX scores were not correlated with RALE scores across the entire cohort (Fig. 5C). Thus, thermal imaging scores did not correlate with the findings on CXR.

## Discussion

**Figure 6:**
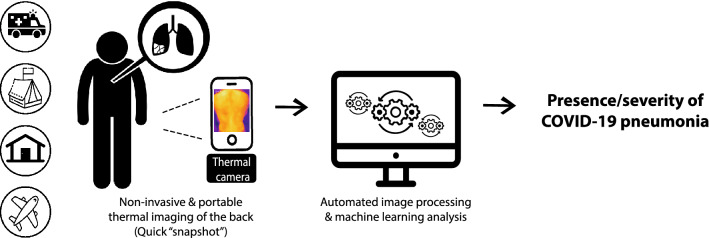
Graphical abstract. A schematic illustration of the research design and potential impact.

Our results show, for the first time, that a hand-held thermal imaging device that connects directly to smartphones can detect individuals infected with SARS-CoV-2 (Fig. 6). Automated thermal image processing of the back yielded two risk scores that demonstrated up to 92% sensitivity in detection of COVID-19.

Portable thermal imaging has several key advantages over currently available diagnostic alternatives for COVID-19 patients. Primarily, this method is well suited for out-of-hospital settings and low-income regions that have limited access to more advanced imaging modalities such as ultrasound, CT, and MRI. Our thermal imaging technique does not require prolonged training or expertise and is compatible with low-cost thermal cameras, such as the one used in this study (FLIR ONE). Portable thermal imaging could be used to screen suspected individuals for COVID-19 and/or associated pneumonia in out-of-hospital and quarantine settings, as well as in nursing homes, in the field and in home-hospitalization cases. Non-contact thermal imaging does not expose patients to ionizing radiation, does not require disposable reagents, and could become a first-line diagnostic tool for COVID-19, thus aiding in early triage and resource allocation in low-income regions.

Current efforts to use infrared thermography in the fight against the COVID-19 pandemic have focused primarily on measuring absolute body temperature and screening for individuals with fever in crowded settings^4,5^. However, these attempts have demonstrated limited effectiveness and were not shown to have a substantive impact on blocking the spread of COVID-19^4,5^. Our innovative approach to extract advanced texture and shape features from the thermal image could help improve these screening tools and aid in the diagnosis of other end-organ damage associated with COVID-19, such as cardiac dysfunction and acute kidney injury^16,17^. The fact that absolute body temperature did not differ between COVID-19 positive and negative patients in our cohort supports our rationale to focus on temperature distribution across organs of interest, as opposed to standard temperature assessment.

The physiological basis of our findings is not entirely clear. We suggest that the differences in skin temperature distribution across the back, as measured by our advanced texture features FD and SX, may reflect microvascular dysfunction caused by COVID-19^18,19^. The fact that both thermal features were inversely correlated with CRP and D-dimer concentrations supports the concept that these thermal scores reflect elevated systemic inflammation in infected individuals, and especially so in patients with lung injury. Microvascular dysfunction has been associated with abnormal temperature distribution across the skin^20,21^. Microvascular and endothelial dysfunction are also characteristic of COVID-19^18,19^. Thus, thermal imaging might detect micro-angiopathies and endothelial dysfunction in COVID-19 patients and could possibly improve risk stratification of infected individuals.

Our study has several limitations. First, we used relatively small sample sizes. Thus, our results are preliminary and need to be validated in larger study populations. Second, the cutoffs used for our thermal scores to detect COVID-19 and lung injury were optimized for this specific study cohort, and need to be adjusted in future multi-center trials with larger cohorts that are more gender- and ethnically diverse. Future large scale efforts might also improve the relatively low specificity levels of our thermal scores for detection of COVID-19 and lung injury. Furthermore, the combination of this technique with additional new machine learning-based diagnostic tools that were recently developed for COVID-19 detection may present an even higher diagnostic yield^1,22,23^. Finally, data on smoking history and BMI were missing for the majority of the cohort. Smoking status and chronic lung injury might affect thermal imaging findings; this aspect will need to be addressed in future studies. Additional confounding factors that should be examined in future studies include menopausal status, pregnancy, thyroid hormonal status, and the effect of various anti-inflammatory medications. Thermal imaging could be a useful tool to monitor various treatments in COVID-19 patients. It is therefore important for future efforts to determine the effects of these treatments on the thermal profile of different body parts over time.

In summary, we have developed here a non-contact portable thermal imaging tool to detect COVID-19 and lung injury (Fig. 6). This technique could facilitate the screening of large numbers of people in order to detect infected individuals in real time, limit the spread of COVID-19, and aid in the allocation of resources throughout the healthcare system.

## Methods

### Study cohort

We recruited individuals with and without COVID-19 from two medical centers in Israel: the Sheba Medical Center, Ramat Gan, and the Tel Aviv Sourasky Medical Center, Tel Aviv. Healthy individuals were also recruited from the Afeka Tel Aviv College of Engineering, Tel Aviv. The study was approved by the Sheba Medical Center institutional review board (IRB), the Tel Aviv Sourasky Medical Center IRB, and the Afeka Tel Aviv College IRB, and all research was performed in accordance with relevant guidelines/regulations and the declaration of Helsinki. All participants and/or their legal guardians provided verbal (by audio recording) or written informed consent prior to their participation in the study.

The research design and patient selection process are presented in Supp Fig. 2. Briefly, we recruited individuals from three sources: (1) patients admitted to the emergency department (ER) (n = 37); (2) patients hospitalized at the department of confirmed COVID-19 cases (n = 55); and (3) healthy individuals from all three participating institutions (n = 14). Five patients were excluded (Supp Fig. 2).

We included men and women (aged 18-90 years) with suspected pneumonia and/or suspected COVID-19 who had undergone a CXR or CT scan up to one week prior to the day of recruitment. To enroll a wide spectrum of control patients, we also recruited patients without any respiratory symptoms or suspected SARS-CoV-2 infection (Table 1). All patients were enrolled within the institution buildings and were not exposed to the sun before the thermal image acquisition. We excluded patients with any of the following conditions: lung malignancy, rheumatic disease with shoulder or back joint involvement, acute or chronic skin disease of the chest or back, critically ill patients with mechanical ventilation, and those unable to provide informed consent. Furthermore, individuals that performed physical activity up to 12 h before thermal image acquisition were not included in the study.

### Clinical data and classification

We collected participants' medical history and clinical evaluation, along with results of blood tests and CXR/CT scans. The MDClone platform was used to automatically extract multiple demographic and clinical variables from Electronic Health Records^24^.

COVID-19 positive status was defined as presence of a positive SARS-CoV-2 qRT-PCR test during hospitalization or up to 10 days prior to the patient’s visit to the ER.

We classified patients according to the presence of lung injury during the time of thermal image acquisition. Lung injury was defined as any clinical or radiological evidence of pneumonia or acute respiratory distress^12,25^. Specifically, any of the following conditions were defined as lung injury: 1) any pathological findings on CXR up to 1 week before recruitment (including ground glass appearance, consolidation, and linear opacities^12^); 2) hypoxemia (≤ 93% SPO_2_ measured in room air on recruitment day); 3) significant respiratory symptoms on recruitment day (including dyspnea, respiratory rate > 20, shortness of breath, and/or severe cough). Control patients included individuals with no respiratory symptoms or chest abnormalities on imaging (regardless of COVID-19 status).

We acquired follow-up data on all hospitalized patients up to 3 months after imaging, including use of non-invasive oxygen therapy, use of mechanical ventilation, and death.

To compare thermal imaging scores with CXR, we manually reviewed CXRs that were acquired on the same day as thermal image acquisition (n = 30), and assigned RALE scores^15^. The CXRs were reviewed by a certified radiologist who was blinded to the clinical statuses of the patients and thermal imaging scores.

### Thermal imaging

We conducted a pilot study aimed to determine the feasibility of our imaging method. This study included 55 individuals, 7 of whom were COVID-19 positive. We used this pilot sample to develop and calibrate our thermal imaging acquisition process, and began to develop the processing algorithms that we subsequently used in our final blinded study cohort.

We captured thermal images using a FLIR ONE thermal camera device (FLIR Systems, Inc. Wilsonville, OR, USA)^26^. FLIR ONE connects directly to smartphones (both iOS- and Android-operated) and utilizes the following functions: a frame rate frequency of 8.7 Hz, an object temperature range of -20 °C to 120 °C, and thermal sensitivity of 100 mK. The wavelength sensitivity over which the camera interpolates temperature is 8-14 µm, and the emissivity value considered appropriate for accurate human temperature readings was 0.98. The room temperature across the hospital is continuously monitored via centralized air conditioning systems and was kept stable at 22-24 °C. The measured humidity is also kept at a relatively stable range of 40-60%.

The optimal imaging procedure included images of the entire backs of the patients. The patients were asked to remove their top clothing and stand in an upright neutral position (hands down the sides of the body in a standard anatomical position). The angle of the image was kept constant with the camera set parallel to the patient’s back ("posterior view"). Three images of the entire back were captured at a distance of about 80 cm (with the back filling the entire frame of the image). The entire imaging procedure lasts up to 3 min (including removal of clothing and positioning). Representative acquired thermal images are presented in Fig. 1.

### Thermal image processing

Thermal images of the final study cohort (n = 101) were analyzed by researchers who were blinded to the patients' clinical statuses. We extracted shape and texture features from the thermal images. We first tested standard features such as mean, median, skewness, kurtosis, and entropy, but these did not provide sufficient discriminative results. We also tested features that are based on the co-occurrence matrix, for instance: contrast, homogeneity, energy, and correlation^7^; these features did not yield a reliable separation between the different clinical classifications. Therefore, we investigated more advanced approaches and developed features that provide a reliable separation. Two main parameters were defined and selected for downstream analysis: one was the fractal dimension^27^ (FD) of the gradient, and the other was a newly defined feature we called the "sum of extrema" (SX) in the image.

The algorithm starts by reading the original thermal image (in numerical format). For the FD, the temperature matrix is first normalized to the range [0, 1]. The image ROI is manually selected, and then a directional Sobel filter, which differentiates along the vertical axis, is applied to the ROI. The columns of the resultant edge image are then attached one after the other, thus forming a one-dimensional vector, which is treated as a signal whose FD we wish to compute. This FD is computed using Higuchi's algorithm^28^ with Kmax = 15.

For the SX, the temperature range in the matrix is first linearly transformed to the range [0, 1], followed by manual selection of the image ROI. Then, each column of the ROI is averaged and a vector of these average values is formed. The second derivative of this vector is computed, and its absolute values from the entire vector are summed. The obtained sum provides the value of the SX, which is described as:

$$SX={\sum }_{j=1}^{M}\left|\frac{{d}^{2}}{{d}^{2}x}\left\{\frac{1}{N}{\sum }_{i=1}^{N}A\left(i,j\right)\right\}\right|$$

where A(i,j) is the ROI, N is the number of rows in the ROI, and M is the number of columns in the ROI.

### Statistical analyses

All continuous variables are displayed as mean (± standard deviation) for normally distributed variables, or median [interquartile range] for variables with nonnormal distributions. Categorical variables are displayed as the number (%) of individuals within each group.

Specific statistical tests are detailed in the figure legends. In brief, differences between values were tested by a two-tailed unpaired t-test. If values were not normally distributed, we used the non-parametric Mann–Whitney test. To assess associations among categorical variables, we used a Chi-square test. We used a one-way ANOVA with Tukey’s test for multiple comparisons to assess the significance of measurements in more than two groups. Outliers were identified by the ROUT method (Q = 1%).

We used a ROC curve under the non-parametric assumption to calculate the AUC for the continuous variables “FD” and “SX” to diagnose three states: 1) COVID-19 disease status; 2) presence of lung injury; and 3) the composite of COVID-19 status and/or lung injury. Sensitivity, specificity, and positive and negative predictive values were calculated for each classification. Spearman’s correlation test was used to assess the correlation between thermal imaging parameters and patients' biomarkers or RALE scores.

Statistical analyses were performed with SPSS (IBM SPSS Statistics, version 25, IBM Corp., Armonk, NY, USA, 2016), GraphPad Prism version 8.00 (GraphPad Software, La Jolla, CA, USA), and MATLAB software (Mathworks Inc. Natick, MA, USA).

## Supplementary Information

Supplementary Information.

## Data Availability

The anonymized datasets generated during this study are available from the corresponding author upon reasonable request. The raw thermal images of the patients will not be available due to patient privacy regulations.
