# Supplementary material for: Automated processing of thermal imaging to detect COVID-19
Source: Sci Rep. 2021 Sep 1;11:17489. doi: 10.1038/s41598-021-96900-9 (PMC8410809; doi:10.1038/s41598-021-96900-9)
Supplement: Supplementary file 1 — Supplementary Information. [file 41598_2021_96900_MOESM1_ESM.docx]

**AUTOMATED PROCESSING OF THERMAL IMAGING TO DETECT COVID-19**

**SUPPLEMENTARY INFORMATION**

**Supp Figure 1: Lower back region of interest**


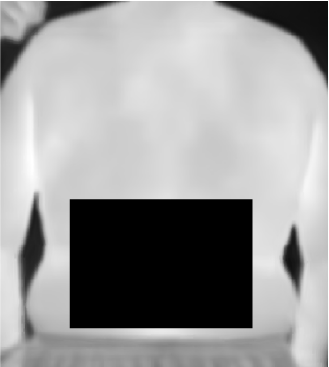


**
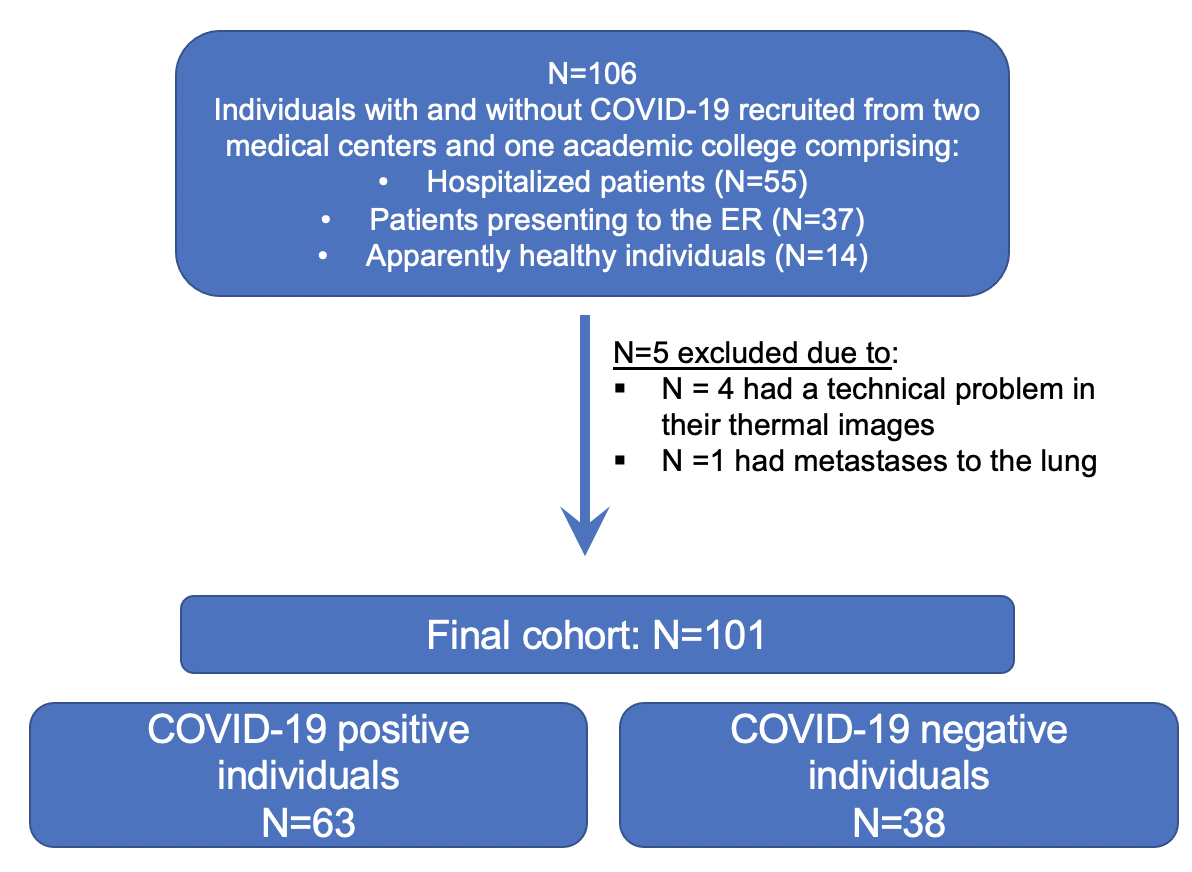
Supp Figure 2: Research design and patient selection**
